# Periprosthetic bone remodeling of short cementless femoral stems in primary total hip arthroplasty: A systematic review and meta-analysis of randomized-controlled trials

**DOI:** 10.1097/MD.0000000000008806

**Published:** 2017-11-27

**Authors:** Shuang G. Yan, Di Li, Shuai Yin, Xingyi Hua, Jian Tang, Florian Schmidutz

**Affiliations:** a Department of Orthopedic Surgery, Physical Medicine and Rehabilitation, University of Munich (LMU), Munich, Germany; b Department of Orthopedic Surgery, The First Affiliated Hospital of Anhui Medical University, Hefei; c Department of Orthopedic Surgery, Changhai Hospital, The Second Military Medical University, Shanghai, China; d BG Trauma Center, Eberhard Karls University Tübingen, Tuebingen, Germany.

**Keywords:** bone remodeling, DEXA, meta-analysis, short stem, total hip arthroplasty

## Abstract

**Background:**

Short-stem total hip arthroplasty (SHA) has been increasingly used in the treatment of hip arthroplasty. However, it is unclear whether there is a superiority of SHA in periprosthetic bone remodeling over standard stem total hip arthroplasty (THA). This meta-analysis of randomized-controlled trials (RCTs) compared the periprosthetic bone remodeling after SHA and THA.

**Methods:**

PubMed and Embase were screened for relevant publications up to May 2017. RCTs that compared periprosthetic bone remodeling with bone mineral density (BMD) changes between SHA and THA were included. Meta-analysis was conducted to calculate weighted mean differences (WMDs) and 95% confidence intervals (CIs) using Stata version 12.0. Quality appraisal was performed by 2 independent reviewers using RevMan 5.3 software and Grades of Recommendation Assessment, Development, and Evaluation criteria.

**Results:**

Seven studies involving 910 patients and 5 SHA designs (Proxima, Fitmore, Microplasty short, Unique custom, and Omnifit-HA 1017) were included for meta-analysis. The pooled data showed no significant differences in the percentage BMD changes in all Gruen zones, with Gruen zone 1 [mean difference (MD) = 11.33, 95% CI, −1.67 to 24.33; *P* = .09] and Gruen zone 7 (MD = 8.46, 95% CI, −1.73 to 18.65; *P* = .10). Subgroup analysis of short SHA stems with lateral flare showed a significant less percentage BMD changes compared with standard THA in Gruen zone 1 (MD = 27.57, 95% CI, 18.03–37.12; *P* < .0001) and Gruen zone 7 (MD = 18.54, 95% CI, 8.27–28.81; *P* < .0001).

**Conclusion:**

The study shows moderate-quality evidence that periprosthetic bone remodeling around the analyzed SHA stems was similar to standard THA stems. However, short SHA stems with lateral flare revealed a moderate- to low-quality evidence for superiority over the standard THA and highlighted the importance of the different SHA designs. Besides, it has to be noticed that despite a similar pattern of periprosthetic bone remodeling, the femoral length where periprosthetic bone remodeling occurs is clearly shorter in SHA. Due to the moderate- to low-quality evidence and the limited stem designs analyzed, the further large-scale multicenter RCTs including the most recent SHA designs are required. However, the current findings should be considered by surgeons for counseling patients regarding total hip replacement.

## 1 Introduction

Cementless femoral stems in total hip arthroplasty provide reliable clinical and radiographic results in the treatment of various hip diseases, such as osteoarthritis, avascular necrosis, and developmental dysplasia.^[1–3]^ However, the bone mineral density (BMD) change in the periprosthetic femoral region due to stress shielding in cementless standard stem total hip arthroplasty (THA) has been well documented.^[4–6]^ These undesirable BMD changes potentially result in early aseptic loosening and revision surgery.^[7,8]^ To address those stress shielding effects and achieve a proximal load transfer in the femoral metaphysis short-stem total hip arthroplasty (SHA) has been developed and is used with increasing numbers.^[9,10]^ Different short-stem designs have been introduced and were classified by Khanuja et al^[11]^ according to their design concept in 4 categories: femoral neck only, calcar loading, calcar loading with lateral flare, and shortened tapered conventional stems.

Minor changes and differences in the THA stem design, such as shortening of the stem length, are able to considerably influence the biomechanical behavior including the periprosthetic bone remodeling. Dual energy X-ray absorptiometry (DEXA) with evaluation of the BMD changes is a well-accepted method to assess the periprosthetic adaptive bone remodeling.^[12]^ The preservation of the metaphyseal bone stock in SHA was reported in several studies by evaluating the BMD changes^[9,13,14]^ and thus might facilitate an exchange from SHA to a standard THA should a revision be necessary. Gasbarra et al^[15]^ confirmed in a prospective study a reduced bone loss in the proximal femur for SHA compared with standard THA. Furthermore, some randomized-controlled trials (RCTs) evaluating the periprosthetic bone remodeling reported that SHA might reduce the periprosthetic bone loss in the proximal femur compared with THA.^[16–18]^ By contrast, other RCTs found no significant differences in BMD changes during periprosthetic bone remodeling between SHA and THA.^[19–22]^ This underlines the controversial discussion in the current literature with no clear evidence of a superiority of SHA in periprosthetic bone remodeling compared to standard THA.

Therefore, we conducted this meta-analysis based on RCTs to investigate the periprosthetic bone remodeling with regard to BMD changes in SHA compared with standard THA.

## 2 Materials and methods

This meta-analysis was conducted on the basis of the Cochrane Handbook recommendations and was reported according to Preferred Reporting Items for Systematic Reviews and Meta-Analyses guidelines.^[23]^ The meta-analysis was registered at PROSPERO (registration number CRD000000). This study involved no direct human trials or animal experiment. Therefore, after contacting the Ethics Committee (Ethical Committee University of the LMU) it was stated that neither a special ethics review nor ethical approval by the ethic committee was needed. Furthermore, due to the retrospective nature using anonymous data, no additional informed consent of the patients was necessary.

### 2.1 Search strategy

We carried out a systematic literature search in PubMed and Embase to identify relevant RCTs from inception to May 2017. The search was performed without restrictions to the language or publication status. We screened databases by using the following terms: (Hip arthroplasty OR Hip replacement) AND (short∗ OR stemless OR metaphyseal) AND (randomized OR controlled clinical trial OR RCT).

### 2.2 Inclusion and exclusion criteria

To be eligible for inclusion, the relevant studies were carefully selected based on the following criteria—Participants: only studies enrolling patients assigned to undergo primary cementless total hip arthroplasty, without age, gender, and racial limitations; Intervention and comparative intervention: clearly documented SHA versus standard THA for total hip arthroplasty, with no restrictions regarding the detailed types of implant; Outcomes: adequate data were provided to calculate the periprosthetic percentage change of BMD (BMD change) postoperatively; and Study design: only RCTs were included.

Studies that did not meet the above inclusion criteria were excluded. Meeting abstracts were excluded due to the unavailable information of the treatment plan, methodological quality, and the risk of bias of the studies.

### 2.3 Data extraction

Two investigators independently extracted the general characteristics and outcomes. In the case of a disagreement not solved by discussion between the 2 investigators, an additional investigator judged the study.

The following information was extracted from each study and entered to a standardized excel file: main author, publication year, original country, inclusion period, number and characteristics of participants, interventions, and follow-ups. Means of BMD changes, standard deviations (SDs), and sample sizes were extracted from original studies. Data presented only in graphs were extracted to numerical values whenever possible, but were included only if 2 reviewers had the consistent results. Unpublished data were obtained by contact with the original investigators and if that failed, calculated with available data. In the absence of SD of percentage BMD changes, the data were retrieved from the mean and SD of BMD at the baseline and final follow-up.

### 2.4 Quality appraisal

We assessed the risk of bias using the Cochrane risk-of-bias tool that included selection bias, performance bias, detection bias, attrition bias, reporting bias, and other bias.^[24]^ The risk of bias summary and graph were obtained from Reviewer Manager 5.3 (RevMan 5.3, Cochrane Collaboration). The strength of evidence for each major outcome was assessed according to the Grading of Recommendations, Assessment, Development, and Evaluation (GRADE) criteria.^[25]^

### 2.5 Statistical analysis

Continuous outcomes (BMD change) at the final follow-up were pooled using the weighted mean difference (WMD) and its corresponding 95% confidence interval (CI). Statistical heterogeneity was quantified using the I^2^ statistic: a value of I^2^ > 50% indicated massive diversity between the studies,^[26]^ and a random-effect model was used; otherwise, a fixed-effect model was applied. Subgroup analyses were performed to detect the source of heterogeneity and investigate the significance of implant design in the periprosthetic BMD changes. Sensitivity analysis was conducted to assess the stability of results and detect the potential source of heterogeneity. Funnel plots and Egger test were created to determine the presence of publication bias.^[27]^ A *P* value of <.05 was considered significant. Data were pooled and graphs were created with the STATA, version 12.0 (StataCorp, College Station, TX).

## 3 Results

### 3.1 Search results

**Figure 1:**
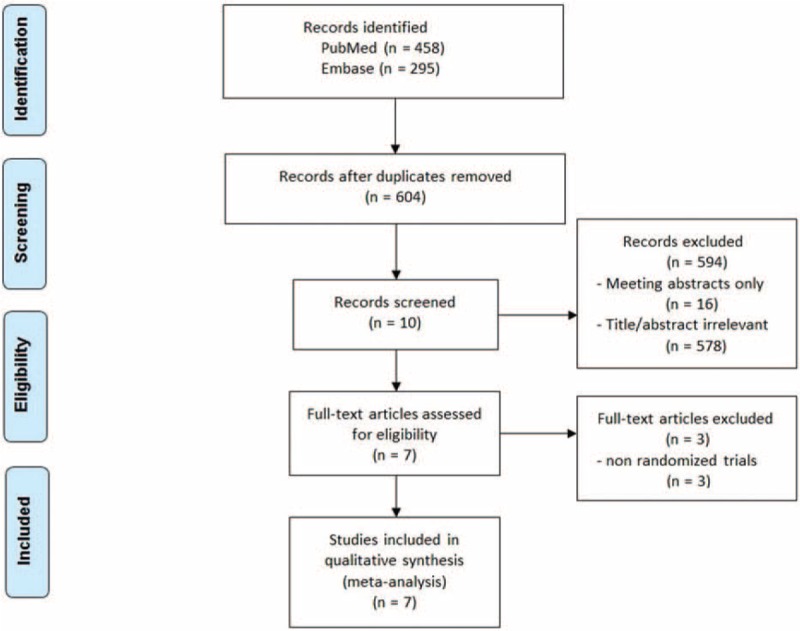
Preferred Reporting Items for Systematic Reviews and Meta-Analyses flowchart for the inclusion of the eligible studies.

Our electronic search identified 753 potentially relevant studies; of which, 149 articles were removed due to duplicate reportage and 594 articles were excluded based on the titles and abstracts that were irrelevant or only meeting abstracts. From the remaining 10 articles, the full text was retrieved and screened, with 3 being excluded as those were non-RCT studies. Finally, 7 randomized-controlled studies fulfilling our inclusion criteria were included in our meta-analysis.^[16–22]^Figure 1 shows the selection process and results from the literature search.

### 3.2 Study characteristics

The characteristics of the included 7 RCTs are summarized in Table 1.^[16–22]^ These studies were published from 2006 to 2016, 5 from Europe^[17,19–22]^ and 2 from Asia.^[16,18]^ The individual sample sizes of these studies ranged from 51 to 400 patients. The studies included a total of 444 patients using SHA, and a total of 466 patients using THA. For the SHA, 4 studies used short stem with calcar loading,^[21]^ shortened conventional,^[19,22]^ and customized designs,^[20]^ while 3 studies used short stem with lateral flare design.^[16–18]^ In all the studies, the BMD (g/cm^2^) data were obtained using DEXA, and the data collected within 1 week after surgery served as the baseline value.^[16–22]^ The comparison of BMD changes was performed in Gruen zones 1 to 7 for 3 studies,^[19–21]^ Gruen zones 1 and 7 for 3 studies,^[16–18]^ and Gruen zones 1, 5, and 7 for 1 study.^[22]^ The mean patient age in the 7 studies was 56.3 years (range 51.8–62.0) with a mean follow-up period of 3.9 years (range 1–11.8). Only 1 study had the follow-up period of more than 10 years,^[18]^ while the remaining studies had the follow-up with maximum 5 years. In order to reduce the heterogeneity, the BMD in that study was extracted at 1 year.^[18]^

**Table 1:**
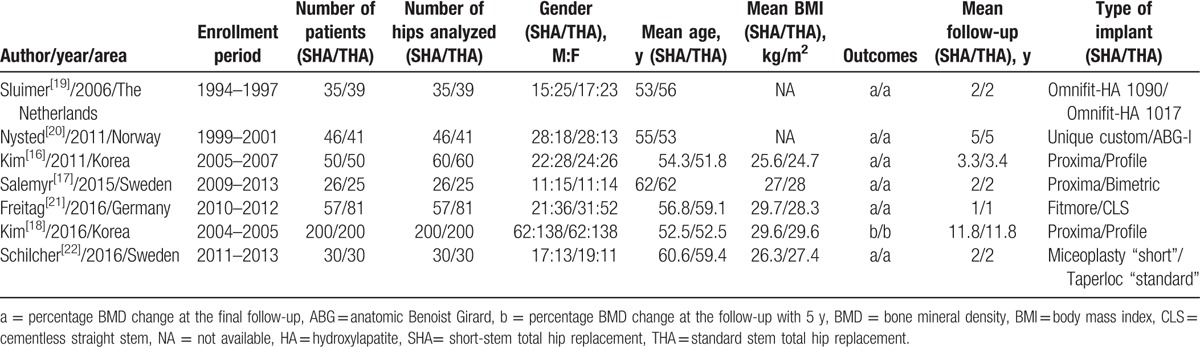
Characteristics of the included studies.

### 3.3 Risk of bias

**Figure 2:**
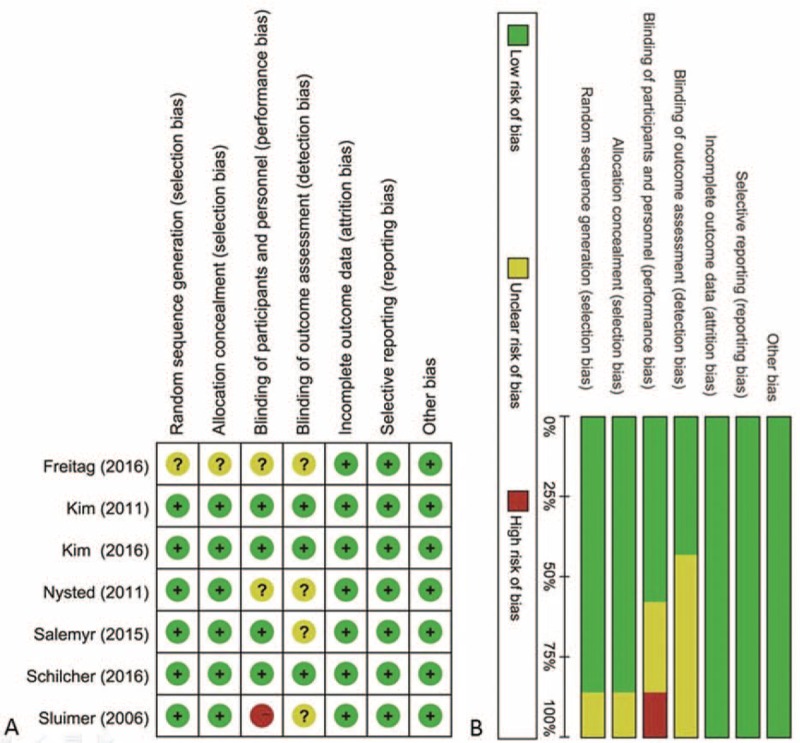
Risk of bias assessment of each included study. (A) Risk of bias summary and (B) risk of bias graph.

In general, the 7 studies were judged as having a low to moderate risk of bias, with the particular risk of bias information of each study given in Fig. 2. An adequate sequence generation method for randomization and allocation concealment was employed by 6 studies,^[16–20,22]^ with information missing for only 1 study.^[21]^ Blinding of participants and personnel was performed in 4 studies,^[16–18,22]^ unclear for 2 studies,^[20,21]^ and the treatment implementation was not blinded by 1 study.^[19]^ Blinding of outcome assessment was performed in 3 studies,^[16,18,22]^ whereas 4 studies did not report this.^[17,19–21]^ All of the included articles displayed a low risk of bias for the incomplete outcomes, selective outcome reporting, and other biases. Patient loss was reported by 4 studies,^[17–19,22]^ however, with a drop our rate below 10%. Table 2 represents the quality of evidence and strength of recommendation according to the GRADE system.^[25]^

**Table 2:**
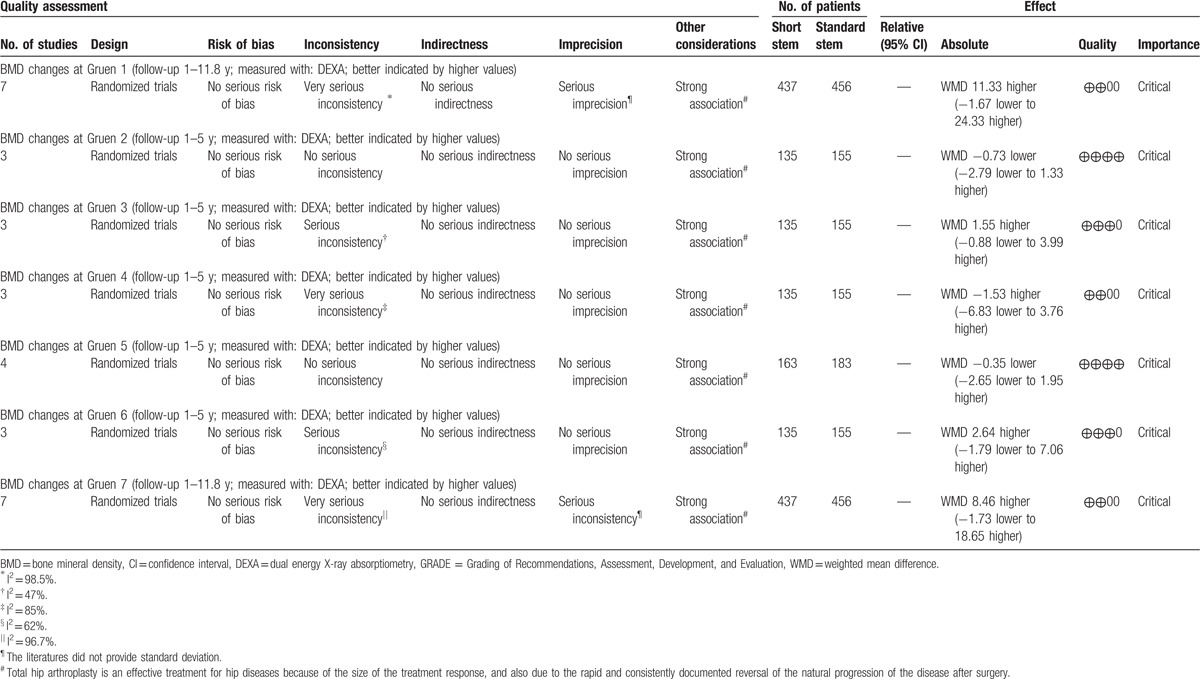
Quality of evidence and strength of recommendations was based on the GRADE system.

### 3.4 Meta-analysis results

#### 3.4.1 BMD change in Gruen zone 1

**Figure 3:**
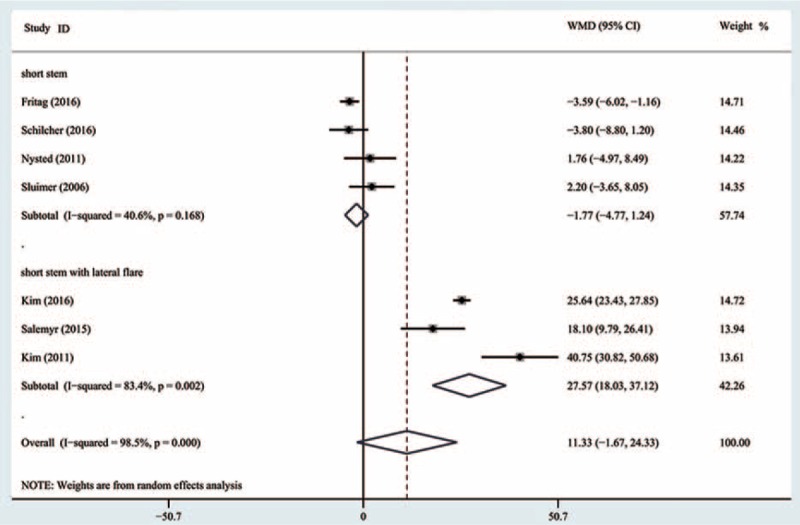
Comparison of the bone mineral density changes in Gruen 1 between cementless short and standard stems in primary standard stem total hip arthroplasty.

Data from 7 studies^[16–22]^ with 910 patients were available for Gruen zone 1. Meta-analysis showed that there was no significantly statistical difference between SHA and THA in the mean BMD change at the final follow-up (WMD = 11.33, 95% CI, −1.67 to 24.33; *P* = .09) (Fig. 3). However, significant heterogeneity was observed among individual trials (I^2^ = 98.5%). Therefore, the random-effects model was used.

Subgroup analysis of the short-stem design revealed no significant difference between SHA and THA in the subgroup of short stem, with the pooled estimate effect −1.77 (95% CI, −4.77 to 1.24; *P* = .25) (Fig. 3) and moderate heterogeneity (I^2^ = 40.6%). A significant difference was observed in the subgroup of short anatomical stem with lateral flare, with the pooled estimate effect 27.57 (95% CI, 18.03–37.12; *P* < .0001) (Fig. 3) and high heterogeneity (I^2^ = 83.4%).

#### 3.4.2 BMD change in Gruen zone 2

**Figure 4:**
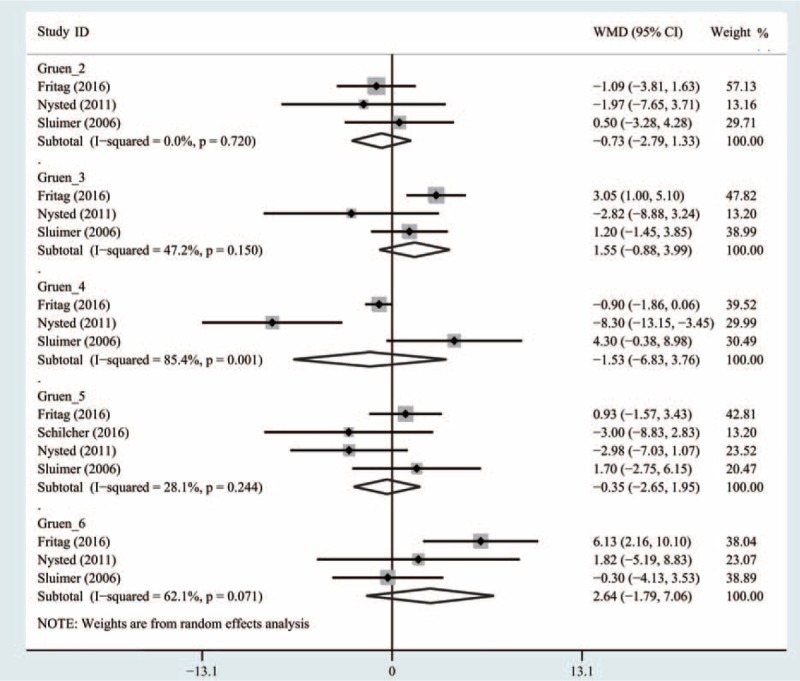
Comparison of the bone mineral density changes in Gruen 2 to 6 between cementless short and standard stems in primary standard stem total hip arthroplasty.

Data from 3 studies^[19–21]^ with 299 patients were available for Gruen zone 2. Meta-analysis indicated that there was no significant difference between SHA and THA in the mean BMD change (WMD = −0.73, 95% CI, −2.79 to 1.33; *P* = .49) (Fig. 4) with low heterogeneity (I^2^ = 0%).

#### 3.4.3 BMD change in Gruen zone 3

Data from 3 studies^[19–21]^ with 299 patients were available for Gruen zone 3. Meta-analysis demonstrated that there was no statistically significant difference between SHA and THA (WMD = 1.55, 95% CI, −0.88 to 3.99; *P* = .21) (Fig. 4) with moderate heterogeneity (I^2^ = 47.2%).

#### 3.4.4 BMD change in Gruen zone 4

Data from 3 trials studies^[19–21]^ with 299 patients were available for Gruen zone 4. Meta-analysis demonstrated that there was no significant difference between SHA and THA (WMD = −1.53, 95% CI, −6.83 to 3.76; *P* = .57) (Fig. 4) with high heterogeneity (I^2^ = 85.4%).

#### 3.4.5 BMD change in Gruen zone 5

Data from 5 studies^[19–22]^ with 359 patients were available for Gruen zone 5. Pooled analysis indicated that there was no significant difference between SHA and THA (WMD = −0.35, 95% CI, −2.65 to 1.95; *P* = .77) (Fig. 4) with low heterogeneity (I^2^ = 28.1%).

#### 3.4.6 BMD change in Gruen zone 6

Data from 5 studies^[19–21]^ with 299 patients were available for Gruen zone 6. Pooled analysis revealed that there was no significant difference between SHA and THA (WMD = 2.64, 95% CI, −1.79 to 7.06; *P* = .24) (Fig. 4) with high heterogeneity (I^2^ = 62.1%).

#### 3.4.7 BMD change in Gruen zone 7

**Figure 5:**
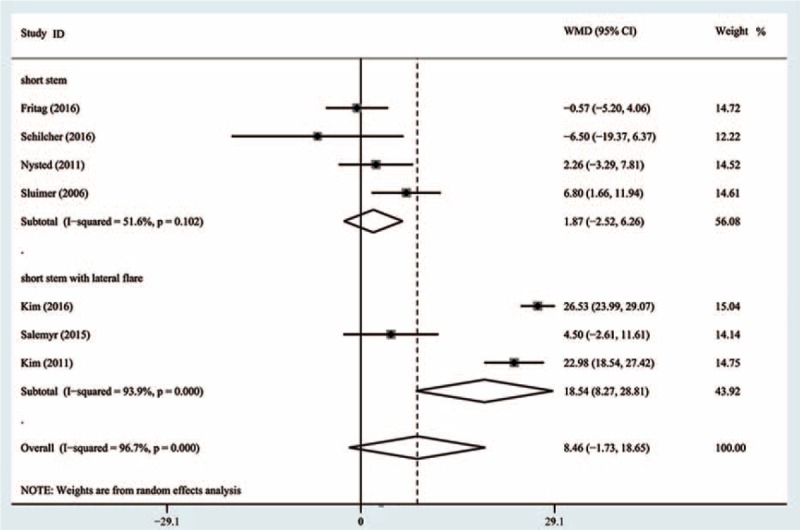
Comparison of the bone mineral density changes in Gruen 7 between cementless short and standard stems in primary standard stem total hip arthroplasty.

Data from 7 studies^[16–22]^ with 910 patients were available for Gruen zone 7. The meta-analysis showed no significant difference between SHA and THA in the mean BMD change at the final follow-up (WMD = 8.46, 95% CI, −1.73 to 18.65; *P* = .10) (Fig. 5) with significant heterogeneity among individual trials (I^2^ = 96.7%).

Subgroup analysis by the design of short stem revealed no significant difference between SHA and THA, with the pooled estimate effect 1.87 (95% CI, −2.52 to 6.26; *P* = .4) (Fig. 5) and moderate heterogeneity (I^2^ = 51.6%). A significant difference was observed in the subgroup of short anatomical stem with lateral flare, with the pooled estimate effect 18.54 (95% CI, 8.27–28.81; *P* < .0001) (Fig. 5) and high heterogeneity (I^2^ = 93.9%).

### 3.5 Publication bias and sensitivity analysis

**Figure 6:**
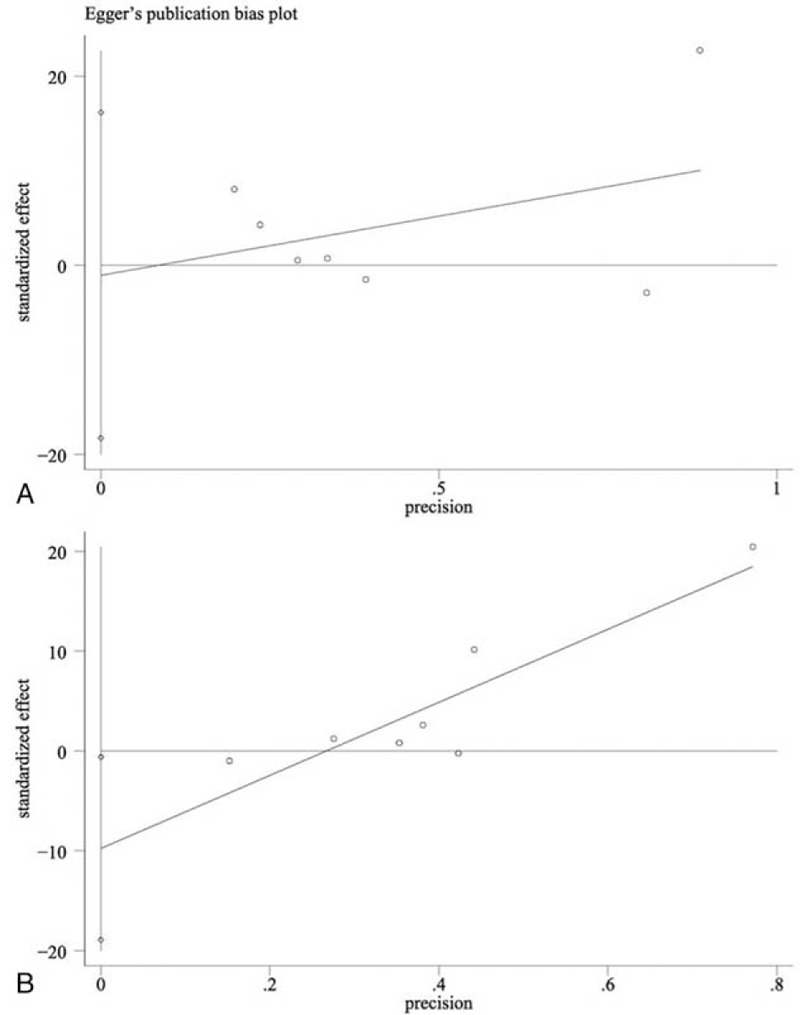
Annotation: funnel plots of publication bias, (A) publication bias for overall BMD change in Gruen 1, *P* = .88 (Egger) and (B) publication bias for overall BMD change in Gruen 7, *P* = .041 (Egger). BMD = bone mineral density.

**Figure 7:**
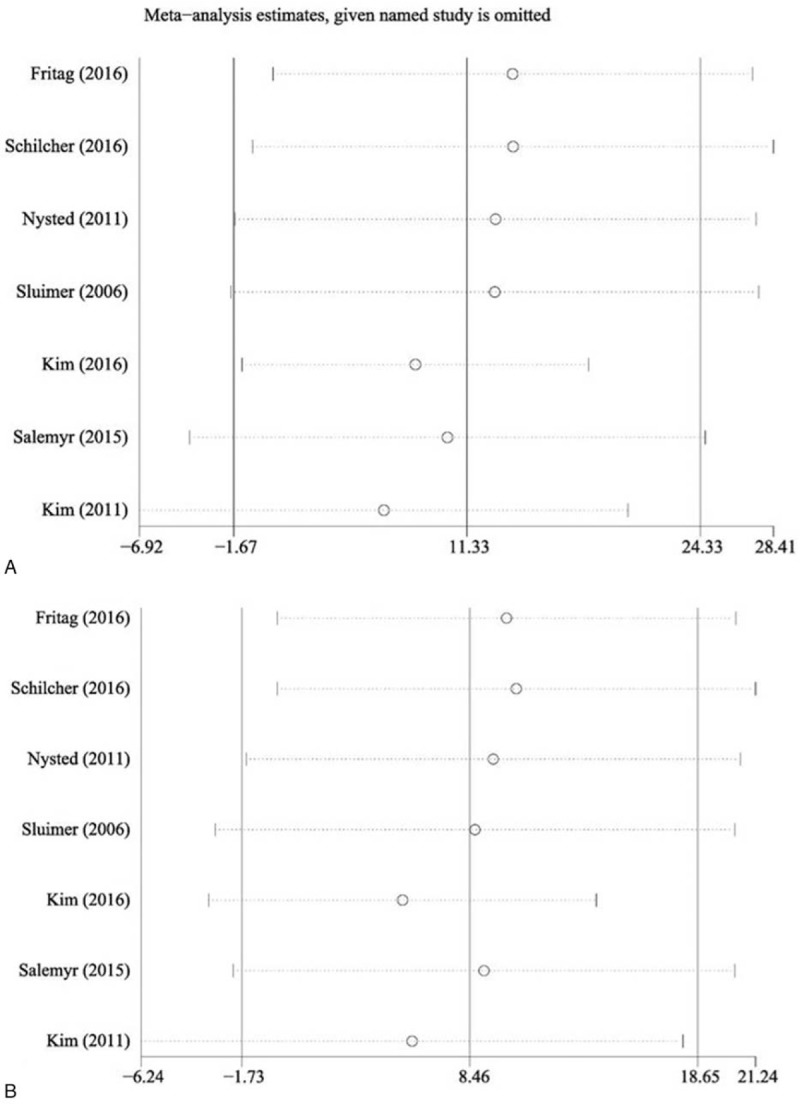
Annotation: forest plot of sensitivity analyses, (A) association between stem length and overall BMD change in Gruen 1 and (B) association between stem length and overall BMD change in Gruen 7. BMD = bone mineral density.

Funnel plot and Egger test were conducted to evaluate publication bias. The results implied that in Gruen zones 1 to 6 all the *P* values of Egger test were >0.05, indicative that publication bias was not evident in these zones, with Gruen zone 7 showing a potential publication bias (*P* = .04). Figure 6 represents Egger publication bias plot in Gruen zones 1 and 7. Sensitivity analysis by the sequential omission of individual studies revealed that none of the included studies affect the final results in Gruen zones 2, 4, 5, and 7, indicating stable and reliable results of the meta-analysis in these zones. A significant change of the overall effect was observed for Gruen zones 1, 3, and 6 in the sensitivity analysis after omitting the study of Freitag et al.^[21]^Figure 7 reports the sensitivity analysis in Gruen zones 1 and 7.

## 4 Discussion

### 4.1 Summary of findings

This meta-analysis of 7 RCTs with a total of 910 patients analyzing the pattern of periprosthetic bone remodeling between SHA and THA revealed no significant difference between both implant designs. The data provide moderate-quality evidence that there was no clear superiority of the evaluated SHA stems in periprosthetic bone remodeling over THA. However, in the subgroup analysis of short-stem SHA with lateral flare, the bone loss in the proximal femur (Gruen zones 1 and 7) was lower compared with the standard THA. This provides moderate- to low-quality evidence that SHA with a short anatomical cementless stem could provide a more physiological pattern of periprosthetic bone remodeling in the proximal femur compared with THA. Besides, it should be noted that the total length of the proximal femur, where periprosthetic bone remodeling occurs, is shorter for SHA stems. These findings are highly relevant for clinicians, healthcare providers as well as researchers, and should also be used for counseling patients requiring total hip replacement.

In this study, the BMD changes in Gruen zones 1 and 7, which are located at the proximal greater trochanter and calcar region,^[28]^ were examined by 7 RCTs.^[16–22]^ For both regions, a reduction in bone substance was observed for both, SHA and THA stems.^[19–22]^ The 3 RCTs that assessed the BMD changes in Gruen zones 2, 3, 4, and 6^[19–21]^ and the 4 RCTs that assessed Gruen zone 5,^[19–22]^ revealed a minor bone loss in these regions for SHA and THA.^[19–22]^ These findings are similar to other clinical studies evaluating the periprosthetic BMD of SHA and THA. Jahnke et al^[13]^ evaluated 40 Metha SHAs after 1-year follow-up and reported a similar pattern of BMD changes, with a reduction in Gruen zone 7 by −11.5% and Gruen zone 1 by −8.4%, while the other regions only showed changes from −1.4% to 6.4%. Similarly, Lerch et al^[5]^ found in a series of 38 Bicontact THAs the highest BMD reduction after 2 years in Gruen zone 1 (−12.9%) and 7 (−8.3%). By contrast, the BMD changes at Gruen 2 to 6 were clearly lower ranging from −6.3% to 6.1%.

Overall, the data of the overall meta-analysis give evidence that there are no clear differences in BMD changes in all the Gruen zones when comparing the SHA to THA. These findings are supported by previous studies reporting that SHA stems could not completely avoid BMD loss in the proximal femoral regions.^[29–31]^ Lazarinis et al^[32]^ reported a substantial loss of BMD in Gruen zone 7 (−28%) after 2 years in a series of 30 collum femoris-preserving (CFP) SHAs. Nevertheless, some contradicting results have also been reported. A prospective study evaluated the periprosthetic bone remodeling of the Fitmore SHA and TMP (Trabecular Metal Primary Stem) THA, and observed a lower BMD loss in all the Gruen zones for SHA compared THA.^[15]^ Similar findings were reported by a previous review which summarized the pattern of periprosthetic bone remodeling that short stems with metaphyseal fixation in SHA allowed a decrease of bone loss in proximal femur compared with THA.^[33]^ However, these results lack a randomization or direct comparison between SHA and THA which may reduce the power of comparability.

Notably, a high heterogeneity in the overall pooled analysis was visible in Gruen zones 1 and 7. In our study, the subgroup analysis by the different classifications of SHA stems could reduce the heterogeneity, showing that stem design is an important factor influencing periprosthetic bone remodeling. Several previous studies confirmed that implants with differing designs produce different patterns of periprosthetic bone remodeling.^[9,34,35]^ Thus it has to be noticed that the most recent generation of SHA, not included in this study, might show a different pattern of periprosthetic bone remodeling. Furthermore, some SHA stems are only a short version of a standard THA stem and result in a similar periprosthetic bone remodeling pattern as the standard THA stem, and therefore have to be clearly distinguished.

This is supported by our subgroup analysis in Gruen zones 1 and 7, which demonstrated that in the subgroup of short anatomical stem with lateral flare, the bone loss in SHA was significantly less than that in THA. However, it has to be noted that some previous studies showed different results. Kim et al^[36]^ conducted a retrospective study and found no significant difference in the periprosthetic BMD changes between short anatomical SHA stems and metaphyseal anchored SHA stems within a 10-year follow-up. Besides, a similar pattern of periprosthetic bone remodeling was observed by a prospective study,^[37]^ which analyzed anatomic and straight stem prostheses. Therefore, further studies are necessary to evaluate the possible advantage of anatomical SHA stems observed in this study and also with respect to the highly variable SHA stem designs.

The observed BMD reduction for both, SHA and THA stems, indicated that the implantation-induced stress shielding cannot be avoided and also altered the proximal loading condition for SHA. Although the shorter stems were designed to prevent distal locking and might facilitate load transfer to the proximal femur,^[9,19,20]^ the evidence from the present study demonstrated a comparable periprosthetic bone remodeling for the evaluated SHA stems with the standard THA stems. Nevertheless, this finding is inconsistent with previous biomechanical studies that reported a reduction of stress shielding in SHA compared with THA.^[38–40]^ The differences can be explained by the different SHA implants evaluated and require further evaluation. Similarly, the observed reduced bone loss around short anatomical SHA in this study demonstrates that design variations in SHA can achieve a reduced periprosthetic bone remodeling. Therefore, further studies with respect to the different SHA designs are necessary before a definite conclusion can be made.

### 4.2 Strengths and limitations of this study

There are several strengths and limitations in this meta-analysis. To the best of our knowledge, this is the first systematic review and meta-analysis that has addressed the topic of periprosthetic bone remodeling in SHA and THA. This study was based on several RCTs from various populations. Besides, this study was strictly operated according to the PRIMA guideline and the level of evidence of results was assessed by the GRADE system.

However, our meta-analysis has some inevitable limitations. First, heterogeneities existed in the length of follow-up and implant design among the included RCTs. Nevertheless, some studies have concluded that the majority of periprosthetic BMD changes following stem implantation occurred in the first postoperative year.^[17,41]^ Second, some included studies did not report the bias of selection, performance, or detection,^[17,19–21]^ which might reduce the reliability of the pooled results. Third, due to the short- to mid-term follow-up in the included studies, the complications resulting from bone remodeling, such as radiolucent line, aseptic loosening, or revision were not assessed. Fourth, due to the unavailable data in the included studies, we did not make the analysis by matching groups with patient-related characteristics, such as gender, age, body weight, and body mass index (BMI). However, those parameters have proved to influence periprosthetic bone remodeling with the BMD progression.^[13,42]^ Fifth, this study only included few of the available SHA designs and mainly not the latest generation of SHA. As those implants feature several design modification, those might give a completely different pattern of periprosthetic bone remodeling. Finally, the number of the trials and implant designs included was relatively small, which therefore requires large-scale multicenter RCTs to draw an inevitable conclusion.

## 5 Conclusions

The results of this meta-analysis demonstrated moderate-quality evidence that in primary total hip arthroplasty the periprosthetic bone remodeling of the analyzed SHA stems was comparable with the standard THA stems. However, there was moderate- to low-quality evidence for a superiority of the anatomical cementless SHA stems, such as the short stem with lateral flare, over the standard THA stem. Besides, compared with standard THA, the shorter length of the SHA stems probably results in a reduced femoral length where bone remodeling processes occur.

Nevertheless, due to the moderate- to low-quality evidence as well as the limited stem designs analyzed, further large-scale multicenter RCTs with longer follow-up are mandatory, especially the influence of the different SHA designs seems to be an important factor and might give substantially differing results. Our current findings should encourage researchers for further RCT studies and also help to assist surgeons to counsel their patients in order to make evidence-based decisions regarding stem selection in primary total hip replacement.
